# What Is Hereditary Transmission?

**Published:** 1856-11

**Authors:** J. J. M. Goss

**Affiliations:** Jackson County, Georgia


### ARTICLE II.

*What is Hereditary Transmission?* By. J. J. M. Goss, M. D., Jackson County, Georgia.

I have often perused voluminous works upon the cause and pathology of the hereditary diseases, to ascertain, if possible, what was meant by the general term of hereditary transmission, but I have generally found but little else than conjecture, or mere assertion, that such and such diseases were transmitted from parent to child. Dr. James Clark, speaking of this subject in his treatise upon Phthisis, says, "that tuberculous-phthisis originates in a morbid state of the constitution." (See Cyc. Prac. Med.) Now, if we admit the disease to originate in a morbid state of constitution, then we must admit that that state exists in direct relation to the form of disease which subsequently becomes developed, and consequently, that that morbid state must be the incipient stage of the disease—the disease itself. Such reasoning explains nothing; it is simply equivalent to the simple declaration, that phthisis exists. If we admit phthisis to originate in a morbid state of the constitution, then these pertinent questions arise: What occasioned this state? And why did it not result in some other hereditary disease, as rheumatism or gout? Neither of these questions admits of more than an exceedingly hypothetical answer; but, under a proper view of the subject, which we will hereafter explain, it follows that the organic forms of the individual determine the form of the disease, under certain geographical and topographical conditions, and any cause of excitement that can produce a derangement of the secretions, may occasion the disease, or it may even spontaneously ensue. We then propose to show that hereditary transmission is not the transmission of a morbid state of constitution, but a defect of organization. If we take a correct view of the relations and functions of the cerebellum and medulla-oblongata, we can very rationally explain what this defect of organization consists in. Combe and other phrenologists state, that the cerebellum presides over the sexual appetites; physiologists state, that it also presides over other functions, viz: muscular motion and desire of urination, defecation, and also, animal sensibility. If the medulla-oblongata be the seat of any one desire, it is certainly that of respiration, because it is found to be large in those persons who manifest the greatest anxiety about this function—namely, consumptives. And, as a general fact, it will be found that those who have the most feeble endowment of the organs of muscular motion and animal sensibility, and the most feeble muscles, have the medulla-oblongata as large as the most muscular men. This relation of the medulla-oblongata and the organs of muscular motion and animal sensibility, clearly shows that the cerebellum has no direct agency in the function of respiration, yet, without its incidental influence, a high endowment of the muscular system, the respiratory function cannot exist. You will not find a well developed thorax associated with a small endowment of the organs of muscular motion. Whenever you find a large endowment of the organs of muscular motion and animal sensibility, especially the former, you may predict the existence of a rheumatic predisposition. We may find syphilitic or mercurial varieties of the disease under contrary circumstances, but never the idiopathic; and from this conclusion, we class gout in the same family. Dr. Wood says, that "there must be a peculiar state of the system predisposing to this form of disease." "There must be a rheumatic diathesis," says he. In what does this diathesis consist? Is it in the blood? then, it is the disease, and not a mere predisposition to it. We would say that it consists in a high endowment of the animal muscles, with considerable sensibility of the skin. Dr. Wood says, "that children under ten years, and adults above sixty, are seldom attacked; and men are more subject than women to this disease." Why are children and women less subject to the disease than men? because they have all parts of the cerebellum less developed than men; and old men are less liable because these functions are on the decline in them. You find active congestion of the brain terminating either in apoplexy, palsy, or epilepsy, when of an idiopathic character, also associated with a high endowment of these organs. You will see, likewise, erysipelatous inflammations, of an original character, attended by a similar endowment. With the same condition of the cerebellum, is a corresponding liability to cutaneous diseases; while those of a contrary organization contract such diseases with difficulty. In proportion to the development of these two organs, will be a liability to, and the progress of, cancerous diseases. You will not find passive congestions of the brain, convulsions, chronic hydrocephalus, chorea sancti viti, tubercular diseases of the lungs, &c., to be unassociated with a feeble endowment of the above named organs. There is one exception to this statement, which is this, when the hemispheres, or that portion of the cerebrum which includes the peculiarly human faculties, preponderates over the balance of it, the vital forces may be very feeble, and yet indicate no tuberculous disposition, because, under such conditions, the venous system preponderates over the arterial—a condition which is incompatible with tuberculosis. It appears, then, that the cerebellum is indirectly connected with the above morbid affections, and we may add, perhaps, many more. Animal sensibility presides over the cutaneous surface; hence, derangement in it may be followed by abnormal actions in the functions of the urinary apparatus, and to these frequently succeed, as sequents, rheumatism, gout, epilepsy, palsy, and other diseases. When the organ of muscular motion is but feebly developed, then we will find passive congestions of the brain, convulsions, hydrocephalus, and consumption, when largely developed, with deficient exercise, we have pulmonary congestions and hemorrhage, or cerebral apoplexy, or gout. And when over-exercised, the kidneys failing to eliminate, the urea that is elaborated in the metamorphosis of muscular tissue, we have rheumatism, epilepsy, or some other disease as the result. With a high endowment of amativeness, and a deficiency of the other two organs of the cerebellum, we find amaurosis to result, also paralysis and imbecility; but with a high endowment of the two, and a deficiency of the posterior lobes of the cerebrum, to furnish motives for such exertions as would expand cerebellar irritation, we have cerebellar apoplexy and mania. There is still another physical condition connected with a relation that is frequently found to exist between the cerebellum and medulla-oblongata. When the medulla-oblongata preponderates over the cerebellum, there will exist a liability to tuberculosis; but when this condition is reversed, there will be an idiopathic disposition to fat, or obesity and dropsy. In these conditions, the thorax is large enough, but the lungs have but little expansive power—such persons never manifest any respiratory action in the facial muscles. According to the above premises, if they be correct, it plainly appears that all the diseases named originate in parts which are exclusively appropriated to the functions of vitality; and by a careful consideration of the temperaments, it can easily be understood how they may so modify the influence of predisposing and exciting causes, as to produce many, and apparently very different, diseases. As for diseases of the vegetative system, we will find them originating in the ganglionic system, and connected, by a sympathetic arrangement, with the anterior extremities of the middle lobes of the cerebrum. And now for the evidence in support of these conclusions. It will be noticed, that those in whom we find a high development of both the medulla-oblongata and cerebellum, will have a large chest, and thoroughly developed animal muscles; their respiration and circulation are promoted by muscular action; their cervical, thoracic, and abdominal muscles aid the diaphragm in accomplishing, fully, the respiratory function: accordingly, in them we find a high development of both the medulla-oblongata and cerebellum. We infer, then, that where the animal muscles are feeble, that respiration is mainly sustained by the diaphragm, consequently, the superior portions of the lungs are but rarely inflated, so becoming feeble from inertia, they soon become the seats of tubercles, for it is admitted that tubercles first invade the superior portions of the lungs. We will admit that this peculiar organic defect is hereditary, but not the disease itself, and would refer the transmission of this defective development of the cerebellum to certain violations of the procreative laws, by which we mean the marriage of individuals who are nearly, or quite the same in temperament. By the last named fact, may be explained the great deterioration, or degeneracy of race, by the intermarriage of relations. Imperfection of organization, resulting in disease and premature death, is often, but very improperly, attributed to an all-wise creator, who never blundered in his work of creation. Disobedience to the laws of organic nature commenced with the dawn of existence, and a greatly enlarged edition of it, with that of civilization, and a want of equilibrium in organization resulted, rendering man incapable of repelling many of the various causes of disease. This defective organization has so multiplied, if not increased, that it may be safely asserted, that hardly half the children, now born, possess an organization adequate to the attainment of three score years. In human society, it has been estimated, that one-fourth the deaths are of children under one year of age; one-half consist of children under five years of age; three-fourths consist of persons under forty-five years of age; six-eighths under sixty; seven-eighths under eighty; and but very few live to be one hundred years of age. Now this difference of longevity, mainly, depends upon a defect in constitution, viz: in that vital force which the original germ received from the parent; as the result of the laws of nature, parents may expect to have secured to them their own vital stamina, their virtues and talents, and, also, an inherent proclivity to their infirmities, vices and crimes. They need never expect to find their offspring sound in constitution, when they themselves are deficient in constitution. They need never look for amiable dispositions in their children, when they themselves do not possess them; children cannot inherit that which the parents do not possess; but they will inherit that which parents do possess, be it good or bad. No man of common sense expects to find pippin apples on crab trees—each tree will produce its kind, and human parents can do no more.

